# Model for predicting short-term mortality of severe sepsis

**DOI:** 10.1186/cc7881

**Published:** 2009-05-19

**Authors:** Christophe Adrie, Adrien Francais, Antonio Alvarez-Gonzalez, Roman Mounier, Elie Azoulay, Jean-Ralph Zahar, Christophe Clec'h, Dany Goldgran-Toledano, Laure Hammer, Adrien Descorps-Declere, Samir Jamali, Jean-Francois Timsit

**Affiliations:** 1 Medical-Surgical Intensive Care Unit, Delafontaine Hospital, 2 rue du Dr Lamaze, 93205 Saint Denis, France; 2 Department of Physiology, Cochin Hospital, Paris Descartes University, Assistance Publique des Hôpitaux de Paris, 27 rue du Faubourg Saint Jacques, 75014 Paris, France; 3 INSERM U823, Epidemiology of Cancer and Severe Illnesses, Albert Bonniot Institute, BP 217, 38043 Grenoble, France; 4 Medical Intensive Care Unit, Hôpital Louis Mourier, 178, rue des Renouillers, 92701 Colombes, France; 5 Medical Intensive Care Unit, Saint Louis Teaching Hospital, 1 rue Claude Vellefaux, 75011 Paris, France; 6 Department of Microbiology, Necker Teaching Hospital, 149, rue de Sèvres, 75743 Paris Cedex 15, France; 7 Medical-Surgical Intensive Care Unit, Avicenne Teaching Hospital, 125, rue de Stalingrad, 93009 Bobigny Cedex, France; 8 Medical-Surgical Intensive Care Unit, Gonesse Hospital, 25 rue Pierre de Theilley, BP 30071, 95503 Gonesse, France; 9 Medical Intensive Care Unit, Albert Michallon Teaching Hospital, Joseph Fournier University, BP 217, 38043 Grenoble cedex 09, France; 10 Surgical Intensive Care Unit, Antoine Béclère Teaching Hospital, 157, rue de la Porte de Trivaux, 92141 Clamart Cedex, France; 11 Medical-Surgical Intensive Care Unit, Dourdan Hospital, 2, rue du Potelet B.P. 102, 91415 Dourdan Cedex, France

## Abstract

**Introduction:**

To establish a prognostic model for predicting 14-day mortality in ICU patients with severe sepsis overall and according to place of infection acquisition and to sepsis episode number.

**Methods:**

In this prospective multicentre observational study on a multicentre database (OUTCOMEREA) including data from 12 ICUs, 2268 patients with 2737 episodes of severe sepsis were randomly divided into a training cohort (n = 1458) and a validation cohort (n = 810). Up to four consecutive severe sepsis episodes per patient occurring within the first 28 ICU days were included. We developed a prognostic model for predicting death within 14 days after each episode, based on patient data available at sepsis onset.

**Results:**

Independent predictors of death were logistic organ dysfunction (odds ratio (OR), 1.22 per point, *P *< 10^-4^), septic shock (OR, 1.40; *P *= 0.01), rank of severe sepsis episode (1 reference, 2: OR, 1.26; *P *= 0.10 ≥ 3: OR, 2.64; *P *< 10^-3^), multiple sources of infection (OR; 1.45, *P *= 0.03), simplified acute physiology score II (OR, 1.02 per point; *P *< 10^-4^), McCabe score ([greater than or equal to]2) (OR, 1.96; *P *< 10^-4^), and number of chronic co-morbidities (1: OR, 1.75; *P *< 10^-3^, ≥ 2: OR, 2.24, *P *< 10^-3^). Validity of the model was good in whole cohorts (AUC-ROC, 0.76; 95%CI, 0.74 to 0.79; and HL Chi-square: 15.3 (*P *= 0.06) for all episodes pooled).

**Conclusions:**

In ICU patients, a prognostic model based on a few easily obtained variables is effective in predicting death within 14 days after the first to fourth episode of severe sepsis complicating community-, hospital-, or ICU-acquired infection.

## Introduction

Severe sepsis remains a leading cause of death in industrialised countries, and the number of deaths caused by sepsis is increasing despite improved survival rates [1,2]. Apart from measures directed to the infectious cause (antibiotics and surgery), the treatment remains chiefly supportive despite many randomised controlled trials [3,4]. Sepsis is a syndrome, not a disease; and many factors explain the variability of outcomes, such as differences in infection sites, causative pathogens, and time and location of infection onset (community, hospital or intensive care unit (ICU)) [1]. This heterogeneity explains that no reliable measures of disease activity have been identified. Attempts to select uniform populations often used ill-defined non-inclusion criteria such as moribund status.

Despite the current tendency to focus on mortality rates after one year or longer, which are highly relevant to cost-effectiveness issues, short-term mortality may be a more appropriate outcome for determining whether new treatments correct the acute effects of severe sepsis. This is because many patients who recover from severe sepsis die later from pre-existing chronic illnesses. Moreover, outcomes and risk factors of patients with severe sepsis vary considerably with the number of episodes and with the time and place (community, hospital or ICU) of acquisition.

The objective of this study was to design a prognostic model for predicting death within 14 days of severe sepsis onset at any time during the first 28 days of the ICU stay. The model was to be based on variables collected at admission and on the day the sepsis episode was diagnosed. Up to four sepsis episodes per patient were included. We evaluated the performance of our model separately in subgroups defined based on the place of infection acquisition. We compared our model with other, widely used scores. Our model may prove useful for designing future studies.

## Methods and materials

### Data source

We conducted a prospective observational study using data entered into a multicentre database (OUTCOMEREA^®^) from November 1996 to April 2007. The database, with input from 12 French ICUs, contains data on admission features and diagnosis, daily disease severity, iatrogenic events, nosocomial infections and vital status. Data for a random sample of at least 50 patients older than 16 years and having ICU stays longer than 24 hours were consecutively entered into the database each year. Each participating ICU chose to perform random sampling by taking either consecutive admissions to selected ICU beds throughout the year or consecutive admissions to all ICU beds over a single month. The contact physicians for the database in the participating ICUs, who are listed in the appendix, are accredited according to French law [5].

### Ethical issues

According to French law, this study did not require patient consent, because it involved research on a database. The study was approved by the institutional review board of the Centres d'Investigation Rhône-Alpes-Auvergne.

### Data collection

Data were collected daily by senior physicians in the participating ICUs. For each patient, the data were entered into an electronic case-report form using VIGIREA^® ^and RHEA^® ^data-capture software (OUTCOMEREA™, Rosny-sous-Bois, France), and all case-report forms were then entered into the OUTCOMEREA^® ^data warehouse. All codes and definitions were established prior to study initiation. The following information was recorded for each patient: age, sex, admission category (medical, scheduled surgery or unscheduled surgery), origin (home, ward or emergency room) and McCabe score [6]. Based on previously reported reproducibility data, the McCabe score was transformed into a dummy variable, that is, 'death expected within five years, yes or no' [7]. Severity of illness was evaluated on the first ICU day using the Simplified Acute Physiology Score (SAPS II) [8], Logistic Organ Dysfunction (LOD) score [9], Sequential Organ Failure Assessment (SOFA) score [10], Mortality Probability models II0 score (MPM0 II score) [11,12], and Acute Physiologic and Chronic Health Evaluation (APACHE) II score [13]. Knaus scale definitions were used to record pre-existing chronic organ failures including respiratory, cardiac, hepatic, renal and immune system failures [13]. Patients were followed until the end of the hospital stay in order to record the vital status 14 days after sepsis onset. For the model, we computed SAPS II and LOD scores based on the data immediately available on admission or on the day (up to 24 hours) before the diagnosis of each episode of sepsis.

### Quality of the database

The data-capture software automatically conducted multiple checks for internal consistency of most of the variables at entry in the database. Queries generated by these checks were resolved with the source ICU before incorporation of the new data into the database. At each participating ICU, data quality was controlled by having a senior physician from another participating ICU check a 2% random sample of the study data.

### Study population

Because diagnostic coding has been found to be unreliable [14], we used parameters collected by our data-capture software to select patients with severe sepsis, defined as systemic inflammatory response syndrome (SIRS) combined with an infectious episode and dysfunction of at least one organ, occurring at or within 28 days after admission to the ICU. We excluded patients with treatment-limitation decisions taken before or on the day of the diagnosis of severe sepsis. At least two of the following criteria were required for the diagnosis of SIRS: core temperature of 38°C or above or 36°C or less, heart rate of 90 beats/min or above, respiratory rate of 20 breaths/min or above, partial pressure of carbon dioxide (PCO_2_) of 32 mmHg or less or use of mechanical ventilation, and peripheral leukocyte count of 12,000/mm^3 ^or above or 4000/mm^3 ^or less. Organ dysfunction was defined as follows: cardiovascular system failure was a need for vasoactive and/or inotropic drugs, and/or systolic blood pressure less than 90 mmHg, and/or a drop in systolic blood pressure by more than 40 mmHg from baseline; renal dysfunction was urinary output of 700 ml/day or less in a patient not previously undergoing haemodialysis for chronic renal failure; respiratory dysfunction was a partial pressure of arterial oxygen (PaO_2_) of less 70 mmHg or mechanical ventilation or a PaO_2_/fraction of inspired oxygen (FiO_2_) ratio of 250 or less (or 200 or less in patients with pneumonia); thrombocytopenia was a platelet count of less than 80,000/mm^3^, and elevated plasma lactate was a lactate level of 3 mmol/L or above. Severe sepsis was defined as sepsis associated with at least one organ dysfunction as described above, and septic shock was defined as sepsis-induced hypotension persisting despite adequate fluid resuscitation together with organ dysfunction. Thus, patients receiving inotropic or vasoactive agents who had organ dysfunction but who were no longer hypotensive were classified as having septic shock [15]. Lengths of ICU and hospital stays were computed starting at ICU admission.

The presence or absence of infection was documented according to the standard definitions developed by the Centers for Disease Control [16]. In addition, quantitative cultures of specimens obtained by bronchoalveolar lavage, protected specimen brush, protected plugged catheter or tracheal aspiration were required to diagnose ventilator-associated pneumonia [17]. Community-acquired infection was defined as infection manifesting before or within 48 hours after hospital admission. Hospital-acquired infection was infection manifesting at least 48 hours after hospital admission but before ICU admission. ICU-acquired infection was diagnosed at least 48 hours after ICU admission. Infection sites were categorised as follows: pneumonia, peritonitis, urinary tract infection, exacerbation of chronic obstructive pulmonary disease, primary bacteraemia (excluding untreated *Staphylococcus epidermidis *bacteraemia), miscellaneous sites (mediastinitis, prostatitis, osteomyelitis and others), and multiple sites. Early effective antibiotic therapy was defined as effectiveness on the causative agent of at least one of the empirically selected antibiotics on the day of the diagnosis of an episode of severe sepsis. Relapse/recurrence was defined as a new episode of severe sepsis with the same microorganism and the same infected organ. New episodes of severe sepsis involving different microorganisms or different organs from the previous episode were classified as separate episodes [18].

### Outcome variable of interest

The outcome variable of interest was death within 14 days after the diagnosis of an episode of severe sepsis (up to four) acquired in the community, hospital or ICU.

We then compared the accuracy of these models with the main ones usual used (SAPS II and APACHE II scores and MPM II_0_).

### Statistical analysis

Our main objective was to develop a patient-based prognostic model that predicted death within 14 days after the diagnosis of the first, second, third or fourth episode of severe sepsis present within 28 days after ICU admission. We randomly allocated two-thirds of the study patients to the training cohort and the remaining one-third to the validation cohort. Up to four episodes of severe sepsis per patient were included, so we conducted a cluster analysis, in which each cluster was composed of one patient with one to four sepsis episodes.

Results were expressed as numbers (percentages) for categorical variables and as medians (quartiles) for continuous variables. Qualitative variables were compared using the chi-squares or Fisher's exact test and continuous variables using the Wilcoxon or Kruskal-Wallis test. A correlation exists between the 14-day outcomes of two consecutive episodes of severe sepsis occurring in the same patient. Consequently, the relation between early death and the study variables was evaluated using generalised estimating equations [19], which are well suited to the analysis of correlated data. We used a logit link function, because the distribution of the outcome variable (14-day mortality) was binary. Correlations between multiple episodes of severe sepsis occurring in the same patient were estimated using Pearson residuals and parameters, according to the maximum likelihood method. We assumed an exchangeable-structure correlation matrix for the data within each cluster. The number of the sepsis episode and the time from admission to the severe sepsis episode were introduced successively into the global model, and the final model that minimised the Akaike information criterion was retained.

Variables associated with early death at the 0.2 level by univariate analysis were introduced in the multivariate model and subsequently selected in order to improve model deviance. The assumption that quantitative variables were linear in the logit was checked using cubic polynomials and graphical methods. In the absence of log-linearity, continuous variables were transformed into qualitative variables according to the slope of the cubic polynomial functions and to the distribution of the variables. A pooled test of clinically relevant two-way interactions was performed on the final model and correlations between selected variables were verified. We checked for potential co-linearity of the variables included in the final model. R values of less than 0.2 were considered acceptable.

Our primary assessment of model performance was goodness-of-fit as evaluated by the Hosmer-Lemeshow statistic and by calibration curves. Lower Hosmer-Lemeshow values and higher *P *values (> 0.05) indicate better fit. We also assessed discrimination (i.e., the ability of the model to separate survivors and non-survivors) using the area under the curve (AUC) of the receiver-operating characteristics (ROC) curve. AUC values greater than 0.80 indicate good discrimination.

The quality of our model was tested separately in community-acquired, hospital-acquired and ICU-acquired sepsis. Then, the final model was evaluated in the validation cohort and compared with other models (SAPS II scores, APACHE II scores and MPM II_0 _score) using the method of Hanley and McNeil to compare AUC-ROC values [19]. Analyses were computed using the SAS 9.1.3 package (SAS Institute, Cary, NC, USA), R and Medcalc 5.00 (Medcalc, Ghent, Belgium).

## Results

**Figure 1:**
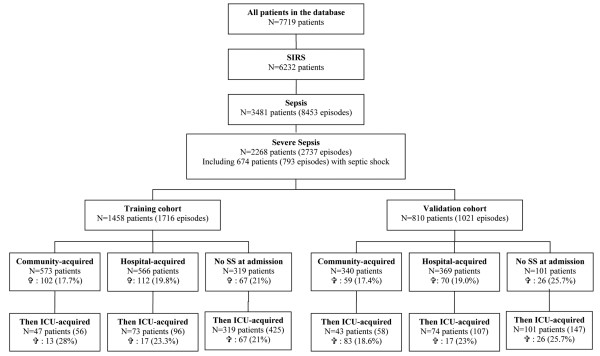
Flow diagram of the 2268 patients with severe sepsis who formed the basis for the study and were identified among the 7719 patients included in the Outcomerea^® ^Database. Data are expressed as counts (number of episodes of severe sepsis (SS)) or percentages. Mortality is defined as death within 14 days after the diagnosis of severe sepsis. community-acquired infection = infection manifesting before or within 48 hours after hospital admission; hospital-acquired infection = infection manifesting at least 48 hours after hospital admission but before ICU admission; ICU = intensive care unit; ICU-acquired infection = infection manifesting at least 48 hours after ICU admission; N = number of patients (number of episode); Sepsis = SIRS with infection; SIRS = systemic inflammatory response syndrome. ✞ Mortality (percentage %).

Among the 7719 patients in the OUTCOMEREA^® ^base, 2268 experienced 2737 episodes of severe sepsis, including 674 patients who had 793 episodes of septic shock. Of the 2268 patients, 1458 patients with 1716 episodes of severe sepsis were included in the training cohort and 810 patients with 1021 episodes of severe sepsis were included in the validation cohort (Figure 1), using a 2:1 randomisation procedure. Characteristics at ICU admission and on the day of severe sepsis onset in 14-day survivors and non-survivors are shown in Tables 1 and 2, respectively. Factors that were significantly associated with early death included worse SAPS II and LOD scores at ICU admission, septic shock (e.g. requiring either inotropic therapy or vasoactive agent support), multiple organ failure (which showed the strongest association) and co-morbidities (immunodeficiency, chronic heart failure, chronic hepatic failure, acute respiratory failure and acute heart failure). On the day of the diagnosis of severe sepsis (Table 2), factors significantly associated with early death included the use of invasive procedures and a need for vasoactive agents and/or inotropic support. *Escherichia coli, Pseudomonas *species, methicillin-resistant *Staphylococcus aureus, Candida *species, bacteraemia and multiple sources of infection were also associated with early death in the univariate analysis.

**Table 1:** Baseline characteristics at ICU admission of 1458 patients with severe sepsis

**Variables at ICU admission**	**Patients alive 14 days after severe sepsis (n = 1177)**	**Patients who died within 14 days after severe sepsis (n = 281)**	***P *value Chi-squared test**
Male gender	725 (61.6)	188 (66.9)	0.10
Age	66 (52 to 76)	69 (56 to 77)	< 10^-2^
Transfer from ward	600 (51)	145 (51.6)	0.85
SAPS II	41 (31 to 53)	59 (44 to 5)	< 10^-4^
LOD	4 (2 to 6)	7 (5 to 10)	< 10^-4^
SOFA	6 (4 to 8)	9 (7 to 12)	< 10^-4^
APACHE II	18 (14 to 22)	24 (20 to 29)	< 10^-4^

**Admission category**	(4 missing)		
Medical	845 (71.8)	207 (73.7)	0.53
Emergency surgery	230 (19.5)	52 (18.5)	0.69
Scheduled surgery	98 (8.3)	22 (7.8)	0.79

**McCabe score**	(4 missing)	(1 missing)	< 10^-4^
1	694 (59)	99 (35.2)	
2	397 (33.7)	134 (47.7)	
3	82 (7)	47 (16.7)	

**Main symptom at admission**			
Multiple organ failure	39 (3.3)	29 (10.3)	< 10^-2^
Shock	367 (31.2)	105 (37.4)	0.05
Acute respiratory failure	384 (32.6)	73 (26)	0.03
Exacerbation of COPD	61 (5.2)	12 (4.3)	0.53
Acute renal failure	50 (4.2)	10 (3.6)	0.60
Coma	140 (11.9)	39 (13.9)	0.36
Trauma	12 (1)	1 (0.4)	0.29
Continuous monitoring	97 (8.2)	7 (2.5)	< 10^-2^
Scheduled surgery	27 (2.3)	5 (1.8)	0.60

**History of immunodeficiency**			
Haematological malignancy	78 (6.6)	29 (10.3)	0.03
Metastatic cancer	59 (5)	25 (8.9)	0.01
AIDS	41 (3.5)	16 (5.7)	0.09
Chemotherapy	90 (7.6)	32 (11.4)	0.04
Steroid therapy	68 (5.8)	28 (10)	0.01
Neutropenia	42 (3.6)	13 (4.6)	0.40

**Co-morbidities (Knaus definitions)**			
Chronic pulmonary failure	198 (16.8)	57 (20.3)	0.17
Immunodeficiency	187 (15.9)	67 (23.8)	< 10^-2^
Chronic heart failure	142 (12.1)	48 (17.1)	0.02
Chronic hepatic failure	52 (4.4)	35 (12.5)	< 10^-2^
Chronic renal failure	29 (2.5)	15 (5.3)	0.01
Exactly one chronic illness	405 (34.4)	136 (48.4)	< 10^-4^
Two or more chronic illnesses	94 (8.0)	42 (15.0)	< 10^-3^
Diabetes mellitus	88 (7.5)	26 (9.3)	0.32

ICU stay (days)	11 (6 to 23)	8 (4 to 12)	< 10^-4^
Hospital stay (days)	33 (19 to 57)	11 (6 to 17)	< 10^-4^

**Type of acquisition of first episode of severe sepsis**			0.46
Community-acquired	471 (40)	102 (36.3)	
Hospital-acquired	454 (38.7)	112 (39.9)	
ICU-acquired	252 (21.4)	67 (23.8)	

APACHE II = Acute Physiologic and Chronic Health Evaluation II; COPD = chronic obstructive pulmonary disease; ICU = intensive care unit; LOD = Logistic Organ Dysfunction; SAPS II = Simplified Acute Physiology Score II; SOFA = Sequential Organ Failure Assessment.

**Table 2:** Baseline characteristics of the 1458 patients in the training cohort, on the first day of severe sepsis

**Variables on the day with severe sepsis**	**Number of episodes of severe sepsis in patients alive 14 days after severe sepsis (n = 1367)**	**Number of episodes of severe sepsis in patients who died within 14 days after severe sepsis (n = 349)**	***P *value Chi-squared test**
**Organ dysfunctions based on the LOD score**			
Neurological	386 (28.2)	155 (44.4)	< 10^-4^
Cardiovascular	590 (43.2)	237 (67.9)	< 10^-4^
Renal	1052 (77)	316 (90.5)	< 10^-4^
Haematological	174 (12.7)	73 (20.9)	< 10^-4^
Hepatic	199 (14.6)	98 (28.1)	< 10^-4^

**Procedures**			
Vasoactive and/or inotropic drugs	681 (49.8)	249 (71.3)	< 10^-4^
Mechanical ventilation	943 (69)	299 (85.7)	< 10^-4^
Arterial catheter	367 (26.8)	142 (40.7)	< 10^-4^
Central catheter	769 (56.3)	266 (76.2)	< 10^-4^
Swan catheter	70 (5.1)	48 (13.8)	< 10^-4^
At least one intravascular catheter	817 (59.8)	278 (79.7)	< 10^-4^
Urinary tract catheter	1081 (79.1)	311 (89.1)	< 10^-4^

**Treatments on the first day of severe sepsis**			
Corticosteroid	350 (25.6)	107 (30.7)	0.06
Antibiotic	1190 (87.1)	294 (84.2)	0.17
Extra-renal replacement therapy	68 (5)	51 (14.6)	< 10^-4^
Early effective antibiotic therapy	1036 (75.8)	250 (71.6)	0.11

**Microorganism**			
*Escherichia coli*	170 (12.4)	60 (17.2)	0.02
*Streptococcus pneumoniae*	104 (7.6)	22 (6.3)	0.41
*Pseudomonas *species	153 (11.2)	52 (14.9)	0.06
*Staphylococcus aureus*	173 (12.7)	44 (12.6)	0.98
Methicillin-resistant *S. aureus*	53 (3.9)	23 (6.6)	0.03
Methicillin-susceptible *S. aureus*	120 (8.8)	21 (6)	0.09
*Candida *species	42 (3.1)	20 (5.7)	0.02
*Enterococcus *species	124 (9.1)	41 (11.7)	0.13
*Acinetobacter baumannii*	14 (1)	3 (0.9)	0.78
Other Gram-positive	110 (8)	22 (6.3)	0.27
Multiple organisms	162 (11.9)	37 (10.6)	0.52
Resistant organisms	95 (6.9)	33 (9.5)	0.05
Unknown	581 (42.5)	124 (35.5)	0.02

**Site of infection**			
Pneumonia	668 (48.9)	171 (49)	0.96
Peritonitis	187 (13.7)	55 (15.8)	0.32
Urinary tract	186 (13.6)	52 (14.9)	0.53
Exacerbation of COPD	127 (9.3)	33 (9.5)	0.92
All forms of bacteraemia	425 (31.1)	134 (38.4)	0.01
Primary bacteraemia	129 (9.4)	34 (9.7)	0.86
Associated bacteraemia	296 (21.7)	100 (28.7)	< 10^-2^
Catheter-related infection	86 (6.3)	22 (6.3)	0.99
Miscellaneous infection sites	136 (9.9)	39 (11.2)	0.50
Multiple infection sites	156 (11.4)	59 (16.9)	< 10^-2^

**Rank of severe sepsis episode**			0.01
One	1177 (86.1)	281 (80.5)	
Two	152 (11.1)	49 (14)	
Three	30 (2.2)	17 (4.9)	
Four	8 (0.6)	2 (0.6)	

COPD = chronic obstructive pulmonary disease; LOD = Logistic Organ Dysfunction.

We determined the best generalised linear model, that is, the model comprising variables that were both readily available and independently associated with early death (Table 3). Among variables collected on the day of diagnosis of severe sepsis, four were associated with an increased risk of early death: worse LOD score, vasoactive and/or inotropic therapy (e.g., septic shock), second episode of severe sepsis compared with the first, and third or fourth episode of sepsis compared with the first. Among infection characteristics entered into the model, only multiple sources of infection significantly increased the risk of early death. Interestingly, the nature of the causative microorganism was not an independent predictor of death. Among variables collected at ICU admission, the following significantly predicted death within 14 days of a sepsis episode: worse SAPS II score, presence of a fatal underlying disease yielding a McCabe score of two or three, presence of one chronic illness, and presence of two or more chronic illnesses. Corticosteroid therapy did not predict early death, even when interactions with septic shock were tested (odds ratio (OR) = 0.99, 95% CI 0.66 to 1.49, *P *= 0.96), and therefore was not included in our model. Absence of early effective antibiotic therapy was associated with death (OR = 0.69, 95% CI 0.53 to 0.91, *P *= 0.01) but was not introduced in the model because this information was not available on the day of severe sepsis.

**Table 3:** Generalised linear model obtained in our study

**Main effect**	**Beta estimate**	**Odds ratio** **95% CI**	***P *value**
Intercept	-4.9419	-	< 10^-4^

**Parameters on the day of severe sepsis**			
LOD (per point)	0.1951	1.22 (1.16 to 1.27)	< 10^-4^
Septic shock	0.3335	1.40 (1.08 to 1.81)	0.01
First episode of severe sepsis	-	-	-
Second episode of severe sepsis	0.2304	1.26 (0.96 to 1.66)	0.10
Third or fourth episode of severe sepsis	0.9719	2.64 (1.71 to 4.08)	< 10^-4^
Multiple sites of infection	0.3734	1.45 (1.04 to 2.03)	0.03
**Variables at ICU admission**			
SAPS (per point)	0.0244	1.02 (1.01 to 1.03)	< 10^-4^
Fatal illness by McCabe Score(score 2 or 3)	0.6749	1.96 (1.43 to 2.70)	< 10^-4^
No chronic illness	-	-	-
Exactly one chronic illness	0.5592	1.75 (1.25 to 2.45)	0.001
Two or more chronic illnesses	0.8084	2.24 (1.39 to 3.62)	0.001

The area under the Receiver-Operating Characteristics curve was 0.822 and the Hosmer-Lemeshow chi-squared test was 8.6 (*P *> 0.05, 8 df), indicating good discrimination and good calibration of the final model in the training cohort. The following variables were tested in the generalized linear model: Logistic Organ Dysfunction (LOD), Sequential Organ Failure Assessment (SOFA), septic shock, high-dose vasoactive drugs (epinephrine and/or norepinephrine > 0.1 γ/kg/min), multiple sites of infection, Simplified Acute Physiology Score (SAPS) II, age, number of chronic organ failures (none, exactly one or two or more), arterial, central venous line or Swan-Ganz catheter, diagnosis at intensive care unit (ICU) admission, year of admission, centre, early effective antibiotic therapy, corticosteroid therapy, male gender, main symptom (multiple organ failure and cardiogenic shock), metastatic cancer, mechanical ventilation, urinary tract catheter, sedation, extrarenal replacement therapy, McCabe score, nature of the microorganism (*E. coli*, *Candida *species and methicillin-susceptible *S. aureus*), infection site and LOD increase from the day before to the day of severe sepsis diagnosis.

To calculate the predicted risk of death for each patient:

- *compute the logit: logit = sum ('Beta estimate' multiplied by value of corresponding parameter)*

- *compute the probability, using the logit: P = (exp (logit)) divided by (1+exp(logit))*

Despite the risk of co-linearity, we considered that LOD on the first day of sepsis and SAPS II at admission could be used in the same model. First, when sepsis was acquired in the ICU, the variables shared by these two scores were not recorded at the same time. Second, using two scores in the same model decreases the loss of information caused by differences in cut-offs. There was no significant co-linearity between our variables (All R values < 0.2).

**Figure 2:**
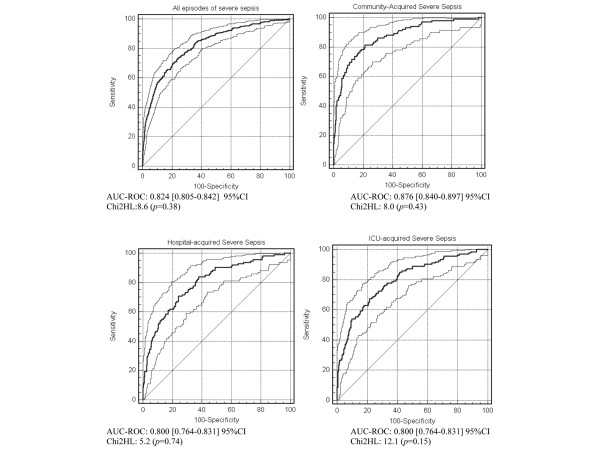
Receiver-Operating Characteristics (ROC) curves and Hosmer-Lemeshow (HL) chi-squared test results of the prediction model in the training cohort. n = 1458 patients, 1716 episodes, according to the type of severe sepsis (community-, hospital- or ICU-acquired). Dashed curves represent 95% confidence intervals (CI) of the area under the curve (AUC) of the ROC curve.

We tested our model in the training cohort in each of the three categories of patients defined by the site of infection acquisition (community, hospital or ICU; Figure 2). In the overall training cohort, the final model exhibited good calibration (Hosmer-Lemeshow (HL) chi-squared, 8.6; *P *> 0.38) and good discrimination (AUC-ROC curve, 0.82). When we confined the analysis to the 573 episodes of community-acquired severe sepsis, the final model showed good calibration (HL chi-squared, 8.0; *P *> 0.43) and discrimination (AUC-ROC curve, 0.87). Validity was satisfactory in the analyses of hospital-acquired and ICU-acquired episodes, with HL chi-squared *P *values greater than 0.05 (0.74 and 0.15, respectively) and AUC-ROC curve values of 0.80 in both groups.

We also evaluated model accuracy for the 1458 first severe sepsis episodes in the training group (n = 1458 patients) versus all subsequent episodes (n = 258, including 56 after community-acquired severe sepsis, 96 after hospital-acquired severe sepsis and 106 after ICU-acquired severe sepsis; Figure 1). AUC was 0.82 for first episodes and 0.82 for subsequent episodes. The difference was not significant according to the Hanley and McNeil test [20]. Moreover, calibration was satisfactory for both groups (HL chi squares *P *> 0.10).

Interestingly, model accuracy was similar for severe sepsis at ICU admission (n = 586, AUC = 0.85) and later in the ICU stay (days 2 to 4: n = 670, AUC = 0.82; days 5 to 7: n = 133, AUC = 0.80; days 8 to 14: n = 200, AUC = 0.80; and days 15 to 28: n = 127, AUC = 0.80). Furthermore, multiple-site infection was not associated with the rank of severe sepsis episode and therefore did not correlate with the number of episodes (*P *= 0.87 by Fisher's exact test).

**Figure 3:**
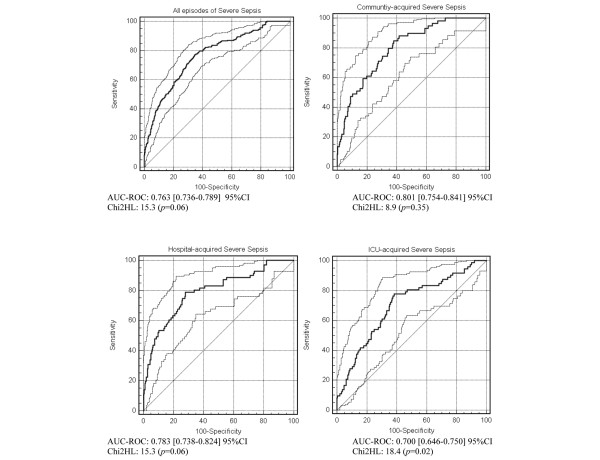
Receiver-Operating Characteristics (ROC) curves and Hosmer-Lemeshow (HL) chi-square test results of the prediction model in the validation cohort. n = 810, 1021 episodes, according to the day of severe sepsis. Dashed curves represent 95% confidence intervals (CI) of the area under the curve (AUC) of the ROC curve.

Performance was slightly lower in the validation cohort (Figure 3). The final model used on all episodes of severe sepsis showed good calibration (HL chi-squared, 15.3, *P *= 0.06) and good discrimination (AUC-ROC curve, 0.76). Results for community- and hospital-acquired infections were satisfactory, with AUC-ROC curve values of 0.80 and 0.79, respectively, and with HL chi-squares *P *values greater than 0.05 in both groups (0.35 and 0.06, respectively). Prediction of early death after ICU-acquired severe sepsis was less accurate, with an AUC-ROC curve of 0.70 but an HL chi-squared *P *value of 0.02. These data are similar to those obtained from calibration curves [See Additional Data File 1, Figure 1].

We also evaluated model performance at different times of the total study period. To this end, we considered three subperiods: 1997 to 2000, 2001 to 2004, and after 2004. Results were similar for these three periods in terms of discrimination and calibration (AUC = 0.802, HL chi-squared = 10.8 for the first period; 0.832 and 4.8 for the second period; and 0.832 and 11.0 for the final period).

**Figure 4:**
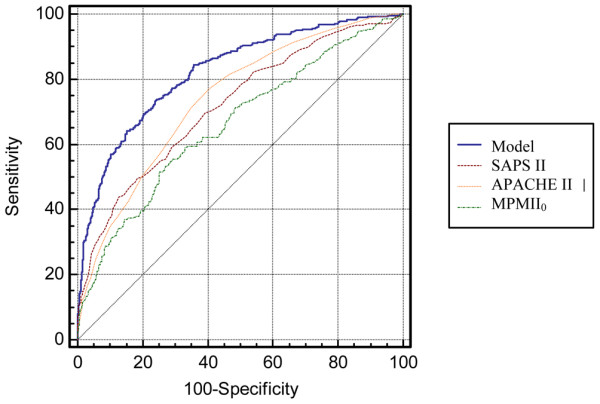
Comparison of our prediction model with other, widely used models. The final study model (blue line) used on all episodes of severe sepsis showed good calibration (Hosmer-Lemeshow (HL) chi-squared 15.3, *P *= 0.06) and good discrimination (area under the curve (AUC)- receiver-operating characteristics (ROC) curve, 0.76). Acute Physiologic and Chronic Health Evaluation (APACHE) II, Mortality Probability models II_0 _(MPM0 II) and Simplified Acute Physiology Score (SAPS) II scores were significantly less accurate than our model, with AUCs of 0.73, 0.66 and 0.72, respectively (P value < 10^-4 ^in all cases), and poor calibration (HL chi-squared *P *values of 0.03, < 10^-4 ^and 0.02, respectively).

Moreover, we compared our model with daily severity scores. APACHE II, MPM II_0 _and SAPS II scores were significantly less accurate than our model, with AUCs of 0.73, 0.66 and 0.72, respectively (P value < 10^-4 ^in all cases), and poor calibration (HL chi-squared *P *values of 0.03, < 10^-4 ^and 0.02, respectively; Figure 4).

## Discussion

We found that predicting death within 14 days after the onset of severe sepsis during the first 28 days in the ICU was feasible in patients with no to three previous episodes of severe sepsis. By adjusting for confounders, we were able to build a predictive model in a training cohort that performed well in the validation cohort. If used in randomised trials, this prognostic model might help to include patients with similar disease severity and to improve adjustment for confounders.

We chose to study short-term mortality, despite the current trend among researchers to focus on long-term mortality [21-23]. Most studies of sepsis used 28-day all-cause mortality as the primary end-point. However, life-limiting disease is a common risk factor for sepsis and may cause death shortly after successful treatment of the septic episode. Early morbidity associated with sepsis is dominated by the side effects of life-supporting interventions (e.g., mechanical ventilation, dialysis and vasoactive agents), whereas delayed morbidity (e.g., neuromuscular weakness, cognitive dysfunction and neuropsychiatric sequelae) is chiefly related to prolonged ICU management. Sepsis is an acute event and its main manifestation, acute organ dysfunction, does not seem to be associated with long-term mortality in patients who survive the original insults [23]. Furthermore, many studies failed to adjust appropriately for treatment-limitation decisions such as do not resuscitate (DNR) given early (less than two days) or later during the ICU stay. Underlying illness is the main reason for DNR orders, which are taken in up to half the patients who die in the ICU [24]. Moreover, treatment-limitation decisions were found to be independently associated with ICU death [25].

Severe infections *per se *are associated with a decrease in life expectancy. In a study that included controls from the general population, sepsis not only caused acute mortality, but also increased the risk of death for up to five years after the septic episode, even after adjustment for pre-existing co-morbidities [26]. The risk of delayed death during the first year was associated with the severity of the septic episode [26]. Several other studies showed that mortality and morbidity rates remained increased for several years among hospital survivors of infection and sepsis [27-31]. However, there is a two-way relation between acute and chronic illnesses. Chronic disease increases the risk of infection and severe sepsis, and survivors of severe sepsis may experience an increase in their burden of chronic disease, which in turn may further elevate the risk for subsequent acute illnesses, thereby initiating a spiral of events that eventually causes death [23]. Therefore, a reasonable hypothesis is that early mortality (e.g., within 14 days) can be ascribed to the severity of acute severe sepsis [32,33] and to the effectiveness of treatment, rather than to underlying chronic illnesses, provided patients with treatment-limitation decisions are excluded, as in our study. Short-term survival may need to be viewed as a surrogate measure, because it is desirable only when followed by long-term survival with an acceptable quality of life. On the other hand, focusing on very long-term mortality, which is extremely relevant to healthcare-cost issues, may mask beneficial effects of drugs used to treat sepsis if the patient dies later on as a result of an underlying chronic illness associated with a risk of sepsis [23]. High death rates due to underlying diseases may explain why many therapeutic trials in patients with severe sepsis failed to detect benefits related to the experimental treatments. Although emphasis is often put on the α risk of false-positive results, the β risk of missing true effects as a result of inadequate statistical power is just as important for the overall population, because false-negative results deprive patients of effective treatments. Therefore, when designing large trials of treatments for severe sepsis, it may be appropriate to select candidate treatments in preliminary trials that use short-term mortality as the primary endpoint.

We found that mortality from severe sepsis could be predicted based on variables associated with the PIRO concept [34] (P: co-morbidities, McCabe; I: multiple-site infection, number of severe sepsis episodes; and R and O: organ dysfunction and vasoactive drug use). These findings are in accordance with a recent report of a PIRO-based score designed to predict 28-day mortality from sepsis, thus focusing on a nearer time horizon than many recent studies evaluating longer term outcome (e.g., longer than three months) [21]. Studies of pneumonia already used 14-day mortality as the primary outcome of interest, to separate the impact of pneumonia from that of co-morbidities or other factors [32,33].

Our study has several limitations. First, the location of the patient before hospital admission was not recorded in the early years of our database. Patients who are chronically in contact with healthcare systems on an outpatient or day-hospital basis (e.g., for chronic dialysis or other chronic treatments) are at risk for severe infection with resistant strains, although they are not admitted [35]. We have been recording this variable since April 2000 and have found that fewer than 5% of patients directly admitted to the ICUs included in the database are recipients of chronic hospital-based outpatient care. Moreover, hospital-acquired infection (in patients transferred to the ICU from other wards) was diagnosed in more than half our patients and was not associated with 14-day mortality (Table 1, *P *= 0.85). Second, calibration as assessed using the HL goodness-of-fit was unsatisfactory (HL chi-squared *P *< 0.05), although discrimination remained good (AUC-ROC = 0.7, 95% CI 0.65 to 0.75) for ICU-acquired severe sepsis in the validation dataset (Figure 3). Third, our model was developed in a single type of healthcare system. External validation studies are needed before the model can be used in countries that have different healthcare systems from the one in France. Finally, it should be borne in mind that the relevance of 14-day mortality to long-term treatment benefits remains to be evaluated. However, our model was clearly superior to widely used models (Figure 4) and may prove helpful for designing and analysing future trials.

## Conclusions

We developed a model for predicting death within 14 days after the diagnosis of the first, second, third or fourth episode of severe sepsis occurring within 28 days after ICU admission. The model is based on a few readily available variables. It may help to evaluate the effectiveness of new drugs or treatment strategies in reversing severe sepsis. In contrast, long-term mortality may be a better marker for the efficacy of treatments directed against sepsis, because recovery from sepsis may be followed by death due to underlying illnesses.

## Key messages

• We developed a model for predicting short-term (14 days) mortality after each episode of severe sepsis, using readily available variables. The model proved very accurate for predicting mortality after one to four severe sepsis episodes in the ICU.

• The model was accurate for community-, hospital- and ICU-acquired episodes of severe sepsis, in both the training and validation cohort (n = 2737 episodes overall).

• This prediction model is designed to predict death directly related to severe sepsis, as opposed to co-morbidities or DNR decisions, which contribute substantially to longer-term mortality rates.

• Our model may help to evaluate the effectiveness of a drug or strategy in severe sepsis, by avoiding type II errors stemming from inadequate statistical power to detect therapeutic effects despite the substantial mortality due to co-morbidities, treatment-limitation decisions and DNR orders.

• In future studies, our model may help to select uniform patient groups for inclusion in clinical trials and to improve adjustment for confounders.

## Abbreviations

APACHE II: Acute Physiologic and Chronic Health Evaluation II; AUC: area under the curve; CI: Confidence Intervals; DNR: do not resuscitate; FiO_2_: fraction of inspired oxygen; HL: Hosmer-Lemeshow chi-squared test; ICU: intensive care unit; LOD: Logistic Organ Dysfunction; MPM II_0_: Mortality Probability models II_0_; OR: odds ratio; PaO_2_: partial pressure of arterial oxygen; PCO_2_: partial pressure of carbon dioxide; ROC: receiver-operating characteristics; SAPS II: Simplified Acute Physiology Score II; SIRS: systemic inflammatory response syndrome.

## Competing interests

OUTCOMEREA is supported by nonexclusive educational grants from Aventis Pharma (France), Wyeth and Pfizer; and by grants from the Centre National de la Recherche Scientifique (CNRS), the Institut National de Recherche Medicale (INSERM) and the Agence Nationale pour la Recherche (ANR). None of these organizations have had input in designing the study reporting the results and publishing it.

## Authors' contributions

CA, AF and JFT participated in the design of the study and writing of the article. All authors participated in data acquisition, data analysis, data interpretation, critical revision of the manuscript for intellectual content and approval of the version submitted for publication. All authors read and approved the final manuscript.

## Supplementary Material

Additional file 1 Word file containing a figure showing calibration curves of both training and validation cohorts. Click here for file

Additional file 2 Word file containing a List of the Members of the Outcomerea Study Group: Scientific committee, Biostatistical and informatics expertise, Investigators and Clinical Research Assistants. Click here for file
