# Supplementary material for: Model for predicting short-term mortality of severe sepsis
Source: Crit Care. 2009 May 19;13(3):R72. doi: 10.1186/cc7881 (PMC2717433; doi:10.1186/cc7881)
Supplement: Additional file 1 — Word file containing a figure showing calibration curves of both training and validation cohorts. [file cc7881-S1.doc]

**Calibration curves**

Overall, calibration was good in the training cohort and satisfactory in the validation cohort, except in the subgroup with ICU-acquired severe sepsis

A- Training dataset

B. Validation dataset
