# Supplementary material for: Model for predicting short-term mortality of severe sepsis
Source: Crit Care. 2009 May 19;13(3):R72. doi: 10.1186/cc7881 (PMC2717433; doi:10.1186/cc7881)
Supplement: Additional file 2 — Word file containing a List of the Members of the Outcomerea Study Group: Scientific committee, Biostatistical and informatics expertise, Investigators and Clinical Research Assistants. [file cc7881-S2.doc]

**APPENDIX**

**Members of the Outcomerea Study Group:**

*Scientific committee:*

Jean-François Timsit (Hôpital Albert Michallon and INSERM U823, Grenoble, France), Pierre Moine (Surgical ICU, Denver, Colorado), Arnaud de Lassence (ICU, Hôpital Louis Mourier, Colombes, France), Elie Azoulay (Medical ICU, Hôpital Saint Louis, Paris, France), Yves Cohen (ICU, Hôpital Avicenne, Bobigny, France), Maïté Garrouste-Orgeas (ICU Hôpital Saint- Joseph, Paris, France), Lilia Soufir (ICU, Hôpital Saint-Joseph, Paris, France), Jean-Ralph Zahar (Department of Microbiology, Hôpital Necker, Paris, France), Christophe Adrie (Department of Physiology, Hôpital Cochin, France), Adel Benali (Microbiology and Infectious Diseases, Hôpital Saint-Joseph, Paris France), Christophe Clec’h (ICU, Hôpital Avicenne, Bobigny, France), and Jean Carlet (ICU, Hôpital Saint-Joseph, Paris, France).

*Biostatistical and informatics expertise:*

Jean-Francois Timsit (Epidemiology of Cancer and Severe Illnesses, INSERM U823, Grenoble, France), Sylvie Chevret (Medical Computer Sciences and Biostatistics Department, Hôpital Saint-Louis, Paris, France), Corinne Alberti (Medical Computer Sciences and Biostatistics Department, Robert Debré, Paris, France), Adrien Francais (Epidemiology of Cancer and Severe Illnesses, INSERM U823, Grenoble, France), Muriel Tafflet (Outcomerea, France); Frederik Lecorre (Supelec, France), and Didier Nakache (Conservatoire National des Arts et Métiers, Paris, France).

*Investigators of the Outcomerea database:*

Christophe Adrie (Department of Physiology, Hôpital Cochin, France), Bernard Allaouchiche (surgical ICU, Hôpital Edouard Herriot, Lyon) Caroline Bornstain (ICU, Hôpital de Montfermeil, France), Alexandre Boyer (ICU, Hôpital Pellegrin, Bordeaux, France), Antoine Caubel (ICU, Hôpital Saint-Joseph, Paris, France), Christine Cheval (SICU, Hôpital Saint-Joseph, Paris, France), Marie-Alliette Costa de Beauregard (Nephrology, Hôpital Tenon, Paris, France), Jean-Pierre Colin (ICU, Hôpital de Dourdan, Dourdan, France), Anne-Sylvie Dumenil (Hôpital Antoine Béclère, Clamart France), Adrien Descorps-Declere (Hôpital Antoine Béclère, Clamart France), Jean-Philippe Fosse (ICU, Hôpital Avicenne, Bobigny, France), Samir Jamali (ICU, Hôpital de Dourdan, Dourdan, France), Christian Laplace (ICU, Hôpital Kremlin-Bicêtre, Bicêtre, France), Thierry Lazard (ICU, Hôpital de la Croix Saint-Simon, Paris, France), Eric Le Miere (ICU, Hôpital Louis Mourier, Colombes, France), Laurent Montesino (ICU, Hôpital Bichat, Paris, France), Bruno Mourvillier (ICU, Hôpital Bichat, France), Benoît Misset (ICU, Hôpital Saint-Joseph, Paris, France), Delphine Moreau (ICU, Hôpital Saint-Louis, Paris, France), Roman Mounier (ICU, Hôpital Louis Mourier, Colombes, France), Etienne Pigné (ICU, Hôpital Louis Mourier, Colombes, France), Carole Schwebel (University hospital A Michallon, Grenoble, France), Jean-Francois Timsit (University hospital A Michallon, Grenoble, France ), Gilles Troché (Hôpital Antoine Béclère, Clamart France), Marie Thuong (Agence de Biomédicine, Saint Denis, France), Dany Golgran-Toledano (CH Gonesse, France), Eric Vantalon (SICU, Hôpital Saint-Joseph, Paris, France), and François Vincent (ICU, Hôpital Avicenne, Bobigny, France).

*Clinical Research Assistants:*

Caroline Tournegros (Hôpital Albert Michallon), Boris Berthe (Hôpital Delafontaine & Avicenne), Silvia Calvino (Hôpital Albert Michallon), Loic Ferrand (Hôpital Albert Michallon), Samir Bekhouche (Hôpital SaintLouis).
